# Metrics for evaluating the stability and reproducibility of mass spectra

**DOI:** 10.1038/s41598-018-37560-0

**Published:** 2019-01-29

**Authors:** E. S. Zhvansky, S. I. Pekov, A. A. Sorokin, V. A. Shurkhay, V. A. Eliferov, A. A. Potapov, E. N. Nikolaev, I. A. Popov

**Affiliations:** 1 Moscow Institute of Physics and Technology, Dolgoprudnyy, Moscow Region, Moscow, Russian Federation; 2 Federal State Autonomous Institution «N.N. Burdenko National Scientific and Practical Center for Neurosurgery» of the Ministry of Healthcare of the Russian Federation, Moscow, Russian Federation; 3 Skolkovo Institute of Science and Technology, Moscow, Russian Federation

## Abstract

In this work, we demonstrate a new approach for assessing the stability and reproducibility of mass spectra obtained via ambient ionization methods. This method is suitable for both comparing experiments during which only one mass spectrum is measured and for evaluating the internal homogeneity of mass spectra collected over a period of time. The approach uses Pearson’s r coefficient and the cosine measure to compare the spectra. It is based on the visualization of dissimilarities between measurements, thus leading to the analysis of dissimilarity patterns. The cosine measure and correlations are compared to obtain better metrics for spectra homogeneity. The method filters out unreliable scans to prevent the analyzed sample from being wrongly characterized. The applicability of the method is demonstrated on a set of brain tumor samples. The developed method could be employed in neurosurgical applications, where mass spectrometry is used to monitor the intraoperative tumor border.

## Introduction

Mass spectrometry methods^1–8^ are widely used for the analysis of biological and medical samples. Ambient ionization mass spectrometry allows rapid analysis of samples to detect various mixture components, pathogens and disease biomarkers^9–12^. In particular, it has been shown that brain tissue samples can be classified based on their lipid profiles; this technique is promising in terms of speeding up and improving the accuracy of analysis of biopsy material obtained during neurosurgery^13–19^. However, in order for mass spectrometry methods to be introduced into routine clinical procedure, all stages of the analysis need to be simplified and automated.

In order to improve molecular profiling methods, one needs to improve the process of analyte microextraction from samples and to optimize the ionization conditions. However, despite the fact that progress has been made in instrumentation to facilitate these improvements, data obtained by molecular profiling is often far from being suitable for subsequent processing, especially when taking into account the need for automated data processing^20–29^. When applying ambient ionization in projects requiring automated identification (classification) of the studied sample, it is essential to develop an approach for unifying the mass spectra, including improving the stability and reproducibility of measurements. This unification method should aim at eliminating scans with any instabilities and those that do not match the characteristics of the sample^30–34^. It should be said that if there is heterogeneity in the sample that is characteristic of the sample itself, it must be taken into account when working with the molecular profile; however, ionization artifacts can lead to errors in the classifiers themselves when they are trained on molecular profile databases. ESI and ESI-based methods, such as DESI and spray-from-tissue, are sensitive, for example, to the homogeneity of the solvent flow and the presence of gas bubbles, similar to how MALDI results are sensitive to the uniformity of MALDI application and matrix crystallization. Use of internal standards is the typical approach for spectra quality control and intensity normalization, but it is hardly applicable to many ambient ionization techniques, especially those designed for *in vivo* sampling.

Despite the fact that, when solving issues related to molecular profiling, the majority of attention is usually given to detecting the presence or absence of one or several biomarkers in the sample, the instability of obtained mass spectra and the low reproducibility of the molecular profile (the peak intensity ratios) can make the results of sample analysis unreliable. This is true when automatic identification is applied to the whole mass spectrometric profile, rather than to a single component of the mass spectrum. Due to the instability of the microextraction and ionization processes, the intensity ratios between the peaks in the spectra can vary significantly between successive measurements of one sample; these differences can become even bigger with different samples and separate measurements. This can then lead to errors in the automatic analysis of the molecular profile^35,36^, which takes into account third-party peaks of the complex biological matrix in addition to the characteristic substances. At the moment, there is no solution that would unify the mass spectra, nor is there a criterion for estimating spectrum quality. This makes it impossible to talk about objectivity, both in terms of data for constructing the classifiers and in terms of the results gained from identifying the type of tissue by its mass spectrum with the help of classifiers. Machine-learning methods and methods of statistical analysis usually require that intra-class variability is less than inter-class variability. Presence of highly variable regions in the spectra contributes to the technical variability, thus leading to a higher level in the overall data variability and rendering the application of statistical methods problematic. Therefore, the use of direct mass spectrometry and, especially, the ionization of tissue samples, requires automated methods for monitoring the stability and reproducibility of spectra.

In this paper, one possible approach to the evaluation and quality control of mass spectra, measured using the ambient ionization methods for the task of classifying biopsy material extracted during a neurosurgical operation, is proposed.

## Methods

### Samples

Tissue samples from primary human brain tumors obtained during the surgery and those removed from patients with a non-tumor pathology (epilepsy) were analyzed. The samples were provided by the N.N. Burdenko NSPCN and analyzed under an approved N.N. Burdenko NSPCN Institutional Review Board protocol. A signed informed consent form, filled out in accordance with the requirements of the local ethical committee specifically noting that all removed tissues can be used for further research, was obtained from all patients before surgery. The study was conducted in accordance with the Helsinki declaration as revised in 2013. All procedures were carried out according to the relevant guidelines and regulations. Samples of tissue removed during surgery were divided into equal parts and half of each sample was subjected to routine hematoxylin and eosin staining and further immunohistochemical analysis, according to which the samples were classified as tumor core (glioblastoma or meningioma) or intact tissue. The other half of each sample was placed in a 0.9% NaCl solution, frozen, and stored at −80 °C until analysis. The samples were defrosted at room temperature, split into smaller fragments, and mass spectra of each fragment were measured. Samples used in this paper include two non-tumorous intact brain tissue samples obtained from two patients with drug-resistant epilepsy during surgery; three samples from patients with glioblastoma (samples with necrosis); three samples from patients with fibroblastic meningioma (WHO grade I).

### Mass spectrometry

The spectra were measured using a high-resolution Thermo Scientific LTQ FT ULTRA mass spectrometer according to the previously described direct spray-from-tissue ionization method, otherwise known as NESI (Needle ElectroSpray Ionization)^37^. The tissue sample (about 2 mm^3^) was placed at the tip of an injection needle (30 mm in length, 0.6 mm inner diameter). Solvent was then pumped through the needle and flowed around the sample (3–5 ul/min HPLC grade methanol (Merck)). Then, a high voltage (6.0 kV in negative mode) was applied to the solvent stream. The spectra were measured in the range m/z 100–1300, with a mass resolution of 56000 at m/z 800. Each measurement lasted for at least five minutes, what corresponds to about 300 scans per mass spectrum measurement in average.

### Data processing

Each mass spectrum was interpreted as an N-dimensional vector. Therefore, the algorithm binned the peaks between m/z 100 and 1300 into 0.01 m/z bins. The binning step was chosen in accordance with the precision of the measurements, which was equal to 2 ppm. This step roughs the data, so the precision of the vector does not exceed the precision of the measurements. A binned spectrum such as this was considered as an N-dimensional vector.

Two characteristics were chosen for comparing the spectra. The first was the Pearson’s r coefficient^38^:

1 $$r=\frac{\sum (X-\bar{X})(Y-\bar{Y})}{\sqrt{{\sum (X-\bar{X})}^{2}}\sqrt{{\sum (Y-\bar{Y})}^{2}}},$$

where *X* and *Y* are vectors of the binned mass spectra, and $\bar{X}=\frac{1}{N}\sum _{i=1}^{N}{X}_{i}$ — mean of *X*, *X*_*i*_ - value of i-coordinate of vector *X*,

The second characteristic was the cosine measure — the cosine of the spectrum angle^38^ (angle between two vectors):

2 $$c=\frac{\sum XY}{\sqrt{\sum {X}^{2}}\sqrt{\sum {Y}^{2}}}$$

The moving median is able to filter out anomalous values, which is not possible with the moving average algorithm, since it takes into account all values including the outlying ones. Thus to remove the influence of the outliers median filtering (moving median^39–41^) was applied. For this, each bin in the smoothed spectra was replaced by the median of corresponding bin values of adjacent scans in smoothing window of size N. Smoothing window was shifted by different steps, compressing the information in the spectra. In this work, we have used smoothing windows of size N = 5, 7, 21 and 51 and steps of one, half of the window and the whole window size. The Pearson’s r coefficient and cosine measure were calculated for the smoothed spectra. All calculations and visualizations were made using a self-written code in the MATLAB software. The source code is available from the authors on request.

**Figure 1:**
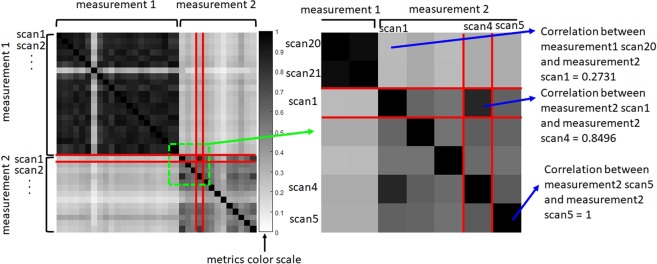
Correlation matrix between two different measurements without smoothing. Dissimilarity metrics of two measurements and inner dissimilarities in each measurement are shown on the left. Scans are grouped by measurements and ordered by time from left to right and from top to bottom within the measurement. The same color scale is used for all following figures in the paper both for Pearson’s r coefficients and cosine measures. Selected fragment is shown on the right at the individual pixel scale.

The results have been visualized as a correlation matrix or cosine measure matrix. Each pixel of the matrix shows the Pearson’s r coefficient between two spectra indexing along the horizontal and vertical axes (Fig. 1). Same colormap and visualization is used for the cosine measure. Scans are grouped by measurements and ordered by the time within each measurement.

## Results and Discussion

**Figure 2:**
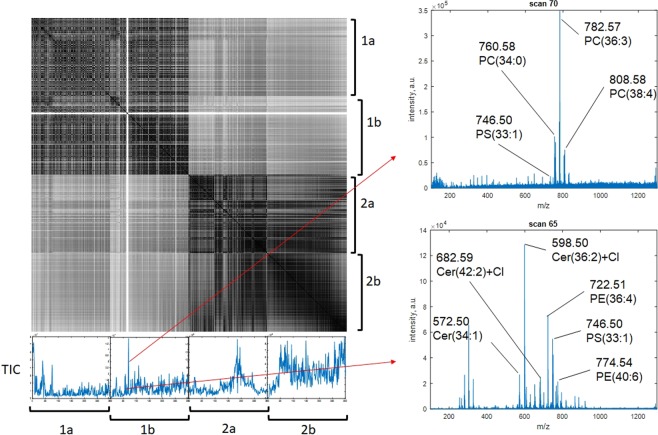
Correlation matrix based on the cosine measure without smoothing. Measurements 1a and 1b correspond to different fragments of one sample classified as a glioblastoma; Measurements 2a and 2b correspond to different fragments of one sample, classified as a meningioma. The total ion current is presented for each measurement, with examples of spectra from two different scans for one of the measurements.

The correlation matrix (Figs 2, S1) demonstrates that spectra of different fragments of the same tissue sample correlate better with each other than with the spectra of fragments of other tissue samples. There are also white lines on the figure, which are associated with electrospray instability (these lines in the correlogram correspond to anomalous local maxima or minima in the total ion current).

### Pearson’s r coefficient and cosine measure

**Figure 3:**
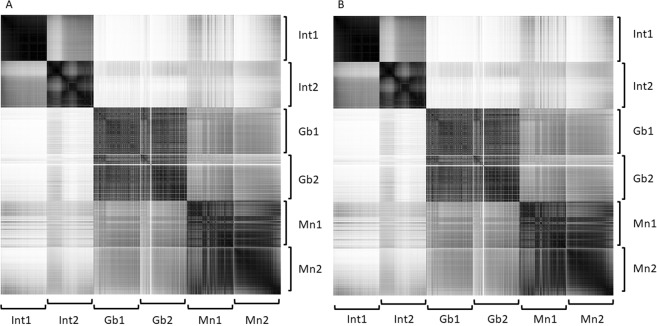
Correlation matrices without smoothing based on Pearson’s r coefficients (**A**) and the cosine measure between vectors (**B**) for two different samples (from different patients) of brain tissue from non-tumor pathology (Int1 and Int2) and two fragments of one sample classified as a glioblastoma (Gb1 and Gb2) and one classified as a meningioma (Mn1 and Mn2).

The Pearson’s r coefficient becomes strictly equal to the cosine measure when the mean is equal to 0, what is evident from formulas (1) and (2). Therefore, since the average values of the peak intensities in a spectrum is close to zero, the cosine measure and the Pearson’s r coefficient between the two spectrum vectors are very similar (Fig. 3). In general, the Pearson’s r coefficient may be negative, although this is unlikely in our case as each scan is a vector of nonnegative values with a mean close to zero. Furthermore, in most scans peaks share their positions so Pearson’s r coefficient even if it is negative has a low absolute value, which can be neglected. At the same time, the cosine measure between vectors with nonnegative coordinates will always be greater than zero. Considering the fact that the cosine measure between the spectrum vectors varies between 0 and 1, as do the Pearson’s r coefficients between vectors, then it can be regarded as a metrics of scan similarity. Calculating the cosine measure requires slightly less computations due to the fact that, for the numerator and denominator, it is not necessary to calculate the mean value of the vector and then subtract it from each vector; therefore, calculating the cosine measure between vectors is preferable when calculating similarities between vectors of high dimensionality.

The visualization of the correlation matrix clearly demonstrates that brain tissues from different non-tumor pathology patients are significantly different from each other (due to natural biological variability), while also being significantly different from tumor tissues. Meanwhile, the tumor samples are quite similar among each of the patients but visibly differ across the diagnoses (Fig. 3). Furthermore, stable changes in molecular profiles occurring during each measurement could be easily found on the correlation matrix. For example, the part of the matrix corresponding to the second meningioma sample demonstrates that the molecular profile changes at the end of the experiment, what could be explained by partial exhausting of the sample during the investigation. However, the last scans of this sample are in good correlation with the initial scans of the first meningioma sample, meaning that these tumor samples are not homogeneous on the molecular level (as the histology examination describes them). Additionally, this demonstrates that NESI is sensitive to sample orientation as exhaustion from the tissue is changed between the parts coming from one sample. Formally the exhaustion is not observed during 5 minutes of spectra measurement. Thus, detection of such cases is vital for effective tissue classification since features of distinctive tissue parts may not be detected in proper proportions in each moment of the measurement and either splitting of spectra into scans or multilabel classification algorithms should be applied.

The fact that the spectrum is normalized to the total ion current, instead of normalizing to the maximum intensity of the spectrum, affects neither the Pearson’s r coefficient or the cosine measure value, as follows from formulas 1 and 2: the cosine measure is independent of the vector length and the Pearson’s r coefficient does not depend on the normalization coefficient. Similarly, with a proportional change in spectrum magnitude, no changes are observed in the calculations of Pearson’s r coefficients or cosine measures. Thus, this technique allows us to automatically take into account as identical those spectra whose intensities are different due to variations in the total ion current values only.

### Spectra smoothing for measurement artifacts filtration

Mass spectrometric profiling of biological samples is usually based on the principle of assigning one specific profile (a mass spectrum) to a sample. In most protocols, this spectrum is simply the sum of the scans accumulated during one measurement. However, it can be improved by discarding the outlier scans that show various anomalies in the measurement process. For this purpose, the scans should be filtered and averaged only afterwards.

For long-term measurements, when multiple mass spectra (scans) are subsequently measured and analyzed, it is necessary to understand whether or not each particular scan should be taken into account. Most statistical and machine-learning algorithms are sensitive to outliers. That is why detection and filtering of outliers is a necessary step for training classifiers. For the detection of outliers the cosine measure between the scan and the smoothed aggregate of all scans in the spectra have to be calculated; then, one can evaluate whether the analyzed scan is a measurement artifact by applying a significance threshold to the calculated value.

The method of median smoothing involves, in a certain sense, adjusting the sensitivity of the obtained spectrum to the signal instability caused, in particular, by the instability of ESI-based sources. This approach makes the stability and reproducibility of this analytical method less sensitive to occasional “bad” scans. In a very unstable spray, a wide smoothing window should be selected for median smoothing to make spectra suitable for automated data processing. In the case when there are no outliers and the stability of the spray and spectra is high enough, the filtering step could be omitted to preserve data for detailed analysis of the ionization process or sample heterogeneity.

**Figure 4:**
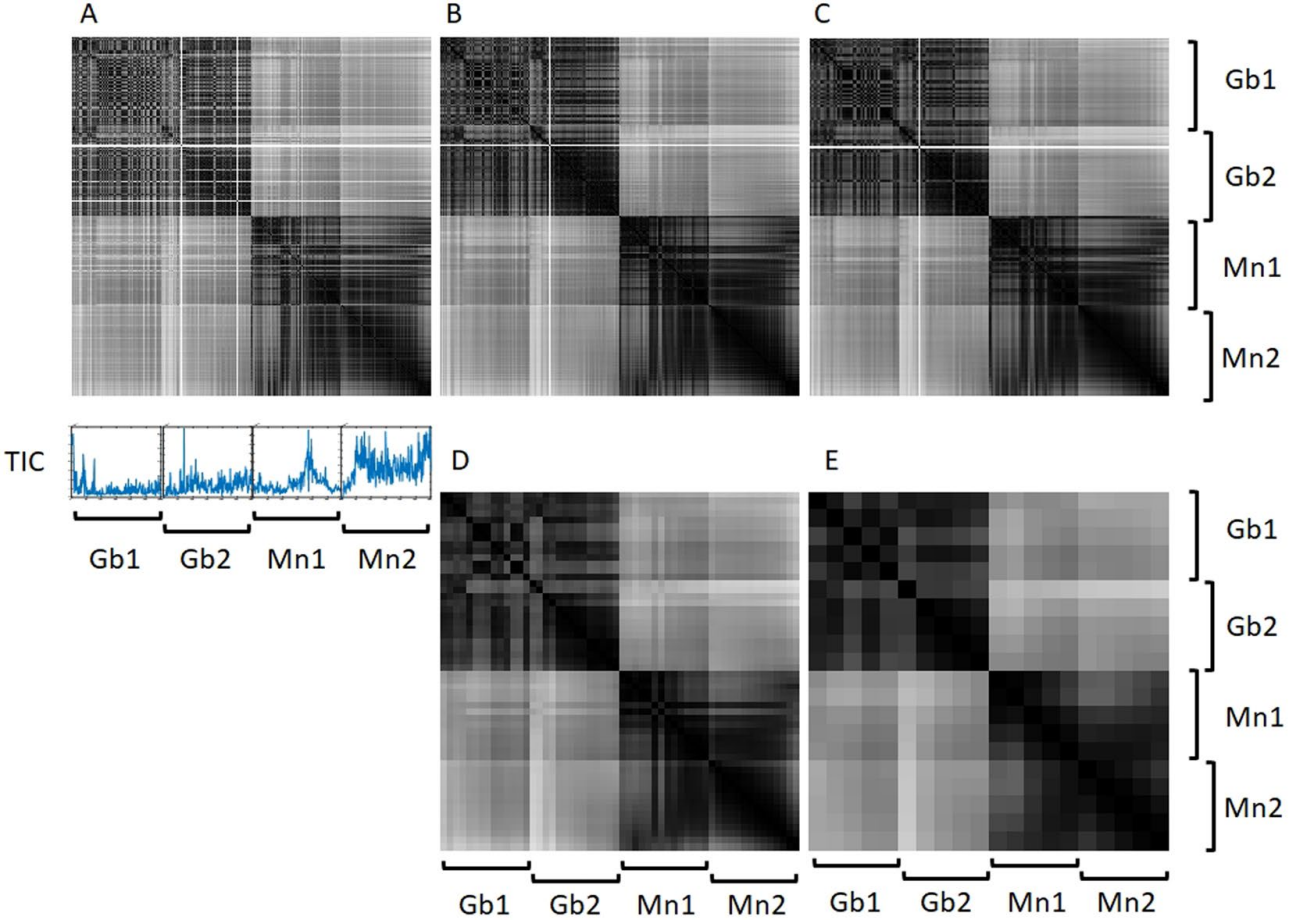
Cosine measure matrices for two fragments of one sample classified as a glioblastoma (Gb1 and Gb2) and two fragments of one sample classified as a meningioma (Mn1 and Mn2). Without smoothing (**A**) and with moving median smoothing with window size equal to 5 (**B**), 7 (**C**), 21(**D**) and 51 (**E**) scans.

Four different windows for the moving median of 5, 7, 21 and 51 scans were considered in order to select the optimal size of the smoothing window (Figs 4, S2). Odd numbers were required to simplify the calculations of median values. The values of 5 and 7 were tested to estimate the number of single spectra with instabilities and to observe their influence on the final result.

As can be seen from Fig. 4B,C, the windows of 5 or 7 scans do not have any fundamental differences; however, they do not filter the outliers (bands with low values) well enough. This is due to the fact that the ICR scans faster if there is no signal (ions in the Penning trap). Thus a more serious smoothing windows were chosen: 51 and an intermediate value of 21 scans. These values correspond to approximately one minute and 30 seconds of averaging, respectively. There is a big difference between the results obtained with windows of 7 and 21, while there is no significant difference between those with 21 and 51. As expected, as the window size increases, the spectra become more similar to each other. The 21-scan anti-aliasing window removes all single outliers, as does the window with 51 scans, but the second requires fewer calculations and makes little difference in the final correlation matrix.

**Figure 5:**
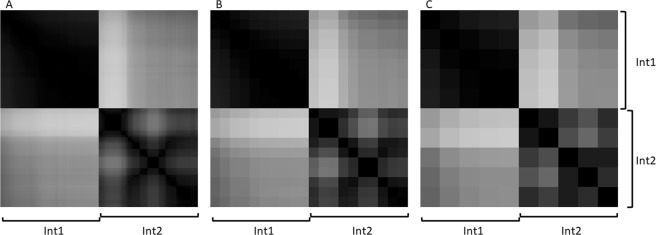
Similarity matrices based on the cosine measure between vectors for two samples classified as a brain tissue from non-tumor pathology (Int1 and Int2), with smoothing over 51 scans using a sliding window shifted by steps of 1, 25 and 51 scans (**A**–**C**, respectively).

We also analyzed the influence of the smoothing window step size on the final results, i.e., the first spectrum was obtained by smoothing over the first 51 scans, and then the subsequent spectra were smoothed over 51 scans beginning from (one + step size) scan. This approach allows to reduce the number of calculations, as well as eliminate anomalous scans and spectra, without losing any information that characterizes the studied sample. Three step values were chosen: 1, 25 and 51 (Fig. 5).

From Fig. 5, a step of 1 is optimal from the point of view of the smoothness of the cosine measure matrix and the absence of rough changes from one smoothed scan to the next. However, it does not reduce the number of calculations. Visually, there is a slight difference between the selected steps. A large smoothing step is optimal for solving problems related to the global grouping of different samples, while a small smoothing step allows the researcher to obtain more detailed information on spectrum changes during the analysis, which may be important when working with highly heterogeneous samples, or in methodological work to determine the optimal parameters and conditions of ambient ionization.

Thus, depending on the task and experimental conditions, it is possible to select the optimal window and smoothing step values. Therefore, for our data and tasks regarding lipid tissue profiling, the use of a moving median with smoothing window of 51 scans is optimal and corresponds to approximately one minute of actual sample measurement. However, this parameter should be optimized with changes in the experimental conditions.

### Metrics application in direct tissue profiling experiments

**Figure 6:**
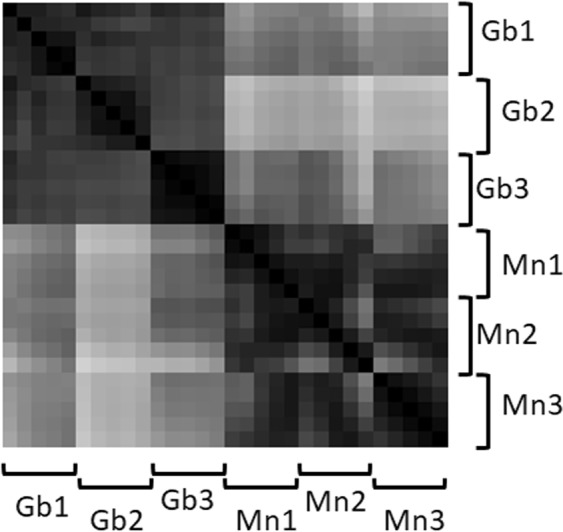
Matrix based on the cosine measure between vectors for three fragments of one sample classified as a glioblastoma (Gb1, Gb2 and Gb3) and three fragments of one sample classified as a meningioma (Mn1, Mn2 and Mn3), with smoothing over 51 scans using a shifting window.

The developed analysis method was used to estimate the selectivity, reproducibility and stability of lipid profiles measured using the NESI method. For these purposes, the data obtained from two samples with different diagnoses, each of the which had been previously divided into three fragments, was analyzed (Fig. 6). It follows from the constructed matrix that the lipid profiles obtained from different fragments are highly reproducible within a single sample and there are large differences between the samples, but for accurate tissue classification statistical analysis and application of classifiers are still required. For this reason metrics calculation with suitably selected averaging parameters allows to select only reliable scans for further automated analysis.

A method for analyzing the stability and reproducibility of mass spectrometric data, obtained in the result of a consecutive accumulation of a large number of mass spectra, has been developed in this study. This method makes allows to filter data for the purpose of further clustering and classification. A method of multidimensional data analysis has been proposed based on the cosine measure between multidimensional vectors and the Pearson’s r coefficient calculations. The proposed analysis method uses median smoothing and facilitates the identification of groups of scans in one measurement, which can be considered as stable and characteristic of the sample. It also eliminates measurement artifacts from consideration, what is especially important for projects dealing with automatic classification of samples based on mass spectrometric profiling methods. The visualization of cosine measure matrices can be used as a simple method for selection of optimal ionization and measurement conditions, in order to evaluate the stability and reproducibility of signals during experimental work using ambient ionization methods.

## Supplementary information

Supplementary information to “Metrics for evaluating the stability and reproducibility of mass spectra” by E.S. Zhvansky, S.I. Pekov, A.A. Sorokin, V.A. Shurkhay, V.A. Eliferov, A.A. Potapov, E.N. Nikolaev, I.A. Popov
