# Supplementary material for: Metrics for evaluating the stability and reproducibility of mass spectra
Source: Sci Rep. 2019 Jan 29;9:914. doi: 10.1038/s41598-018-37560-0 (PMC6351633; doi:10.1038/s41598-018-37560-0)
Supplement: Supplementary file 1 — Supplementary information to “Metrics for evaluating the stability and reproducibility of mass spectra” by E.S. Zhvansky, S.I. Pekov, A.A. Sorokin, V.A. Shurkhay, V.A. Eliferov, A.A. Potapov, E.N. Nikolaev, I.A. Popov [file 41598_2018_37560_MOESM1_ESM.pdf]

Supplementary information to “Metrics for evaluating the stability and reproducibility of mass spectra” by E.S. Zhvansky, S.I. Pekov, A.A. Sorokin, V.A. Shurkhay, V.A. Eliferov, A.A. Potapov, E.N. Nikolaev, I.A. Popov

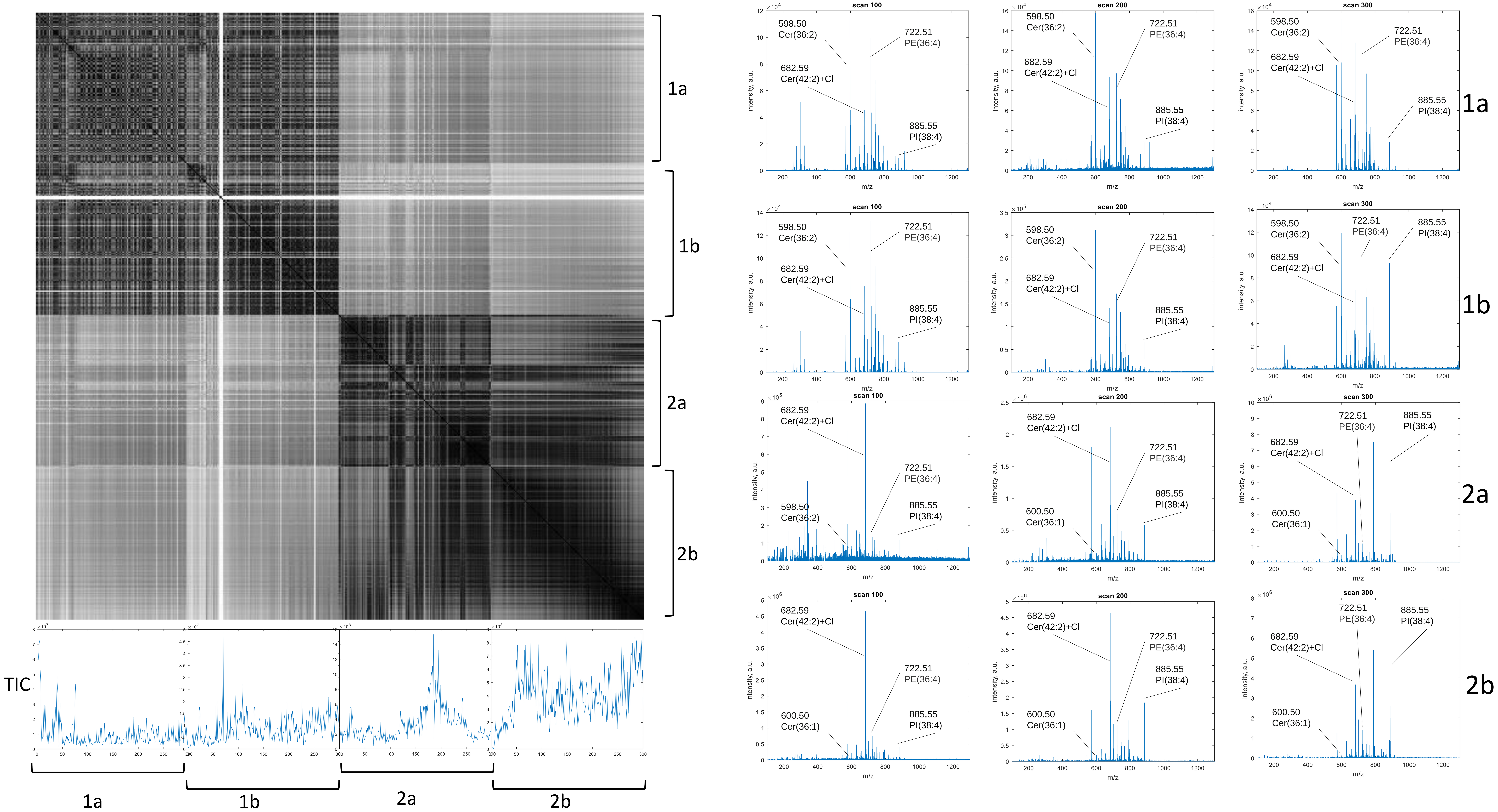

Fig. S1. Correlation matrix based on the cosine measure without averaging. Measurements 1a and 1b correspond to different fragments of one sample classified as a glioblastoma; Measurements 2a and 2b correspond to different fragments of one sample, classified as a meningioma. The total ion current is presented for each measurement, with examples of spectra from two different scans for one of the measurements. Spectra and major peaks' identifications are presented as well as the number of the scan in each measurement in the titles.

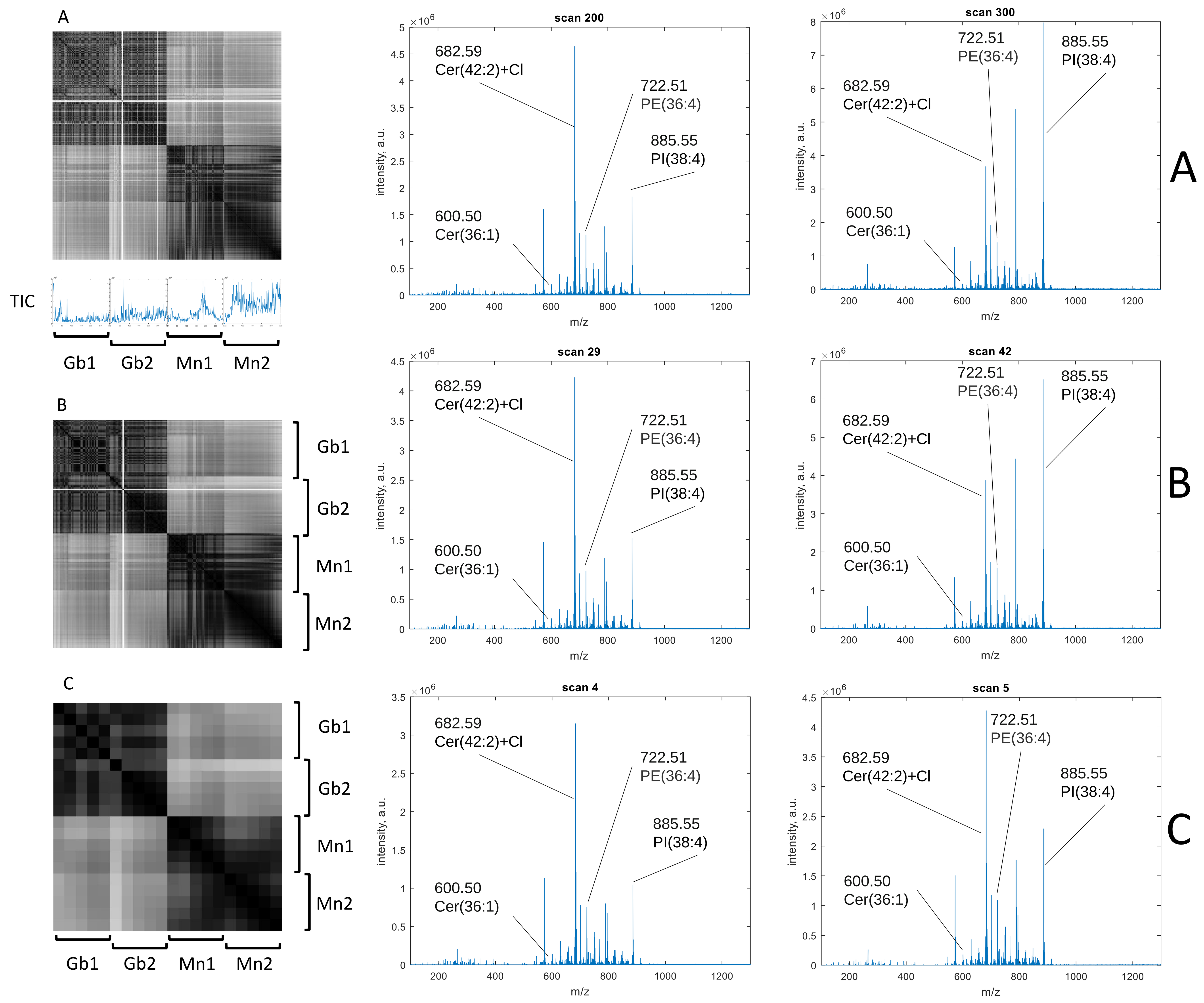

Fig. S2. Cosine measure matrices for two fragments of one sample classified as a glioblastoma (Gb1 and Gb2) and two fragments of one sample classified as a meningioma (Mn1 and Mn2). Without averaging (A) and with averaging for 7 and 51 scans (B and C, respectively) using a moving window. Spectra and major peaks' identifications are presented as well as the number of the scan in each measurement in the titles.
